# The oral–vascular axis: immune mechanisms linking periodontal dysbiosis to systemic vascular pathology

**DOI:** 10.3389/fimmu.2026.1793621

**Published:** 2026-04-17

**Authors:** Jie Yu, Wen-Wen Zhuang, Bo Lei, Rong-Yan Shan, Xi-Meng Wang, Peng Qu, Matthias Hannig, Yong Liu

**Affiliations:** 1 Department of Dentistry, Central Hospital of Dalian University of Technology, Dalian, China; 2 Faculty of Medicine, Dalian University of Technology, Dalian, China; 3 Faculty of Dentistry, Saarland University, Homburg, Germany; 4 Department of Cardiology, The Second Affiliated Hospital of Dalian Medical University, Dalian, China; 5 Department of Cardiology, Central Hospital of Dalian University of Technology, Dalian, China

**Keywords:** atherosclerosis, endothelial dysfunction, periodontal dysbiosis, periodontitis, trained immunity, vascular inflammation

## Abstract

Periodontitis is among the most prevalent chronic inflammatory diseases worldwide and may affect vascular health beyond the oral cavity. Framed within the concept of an oral–vascular axis, this review synthesizes clinical and mechanistic evidence linking periodontal disease with atherosclerotic cardiovascular disease (ASCVD). Epidemiological studies and meta analyses consistently associate periodontitis with higher risks of coronary heart disease (CHD), stroke, and cardiovascular mortality, with modest but reproducible effect sizes that persist after adjustment for traditional risk factors. However, heterogeneous study designs and residual confounding preclude definitive causal inference. Interventional evidence is currently dominated by surrogate endpoints, and event-level cardiovascular benefit from periodontal therapy remains unproven. Mechanistically, chronic periodontal inflammation may influence endothelial function and atherogenesis through interlocking pathways that can be viewed as a spatiotemporal, dual-regulatory network of immunity and metabolism: local dysbiosis and barrier disruption increase systemic access to microbial ligands and vesicular cargo, while systemic immune activation interacts with metabolic remodeling to shape inflammatory set-points and vascular susceptibility. Microbe-derived and host–microbe co-metabolites may further modulate redox balance, inflammatory tone, and vascular homeostasis within this network. We highlight limitations of existing interventional trials, methodological challenges in microbiome- and genetics-based causal inference, and priorities for translational research. Clinically, the oral–vascular axis motivates interdisciplinary exchange and research-facing collaboration that integrates oral health assessment with immune and vascular phenotyping, while recognizing that cardiovascular benefit from periodontal interventions remains investigational and requires event-driven validation.

## 1 Introduction

Periodontal disease is among the most prevalent chronic inflammatory diseases globally. Data from the Global Burden of Disease (GBD 2021) show that its prevalence and disability burden have remained persistently high over the past three decades, making it a long-standing public health challenge across regions and age groups (1). At the same time, the oral cavity is not an isolated organ but an important mucosal ecological niche that contributes to systemic homeostasis. With the development of molecular medicine and multi-omics technologies, links between the oral ecosystem and systemic diseases are being increasingly recognized (2).

Vascular diseases occupy a central position among systemic disorders. Cardiovascular disease (CVD) has long been the leading cause of death worldwide, accounting for approximately 19.8 million deaths in 2022, about one-third of all global deaths (3, 4). This implies that any chronic inflammatory source capable of perturbing vascular homeostasis may carry major public health implications. In recent years, a growing body of epidemiological and clinical studies has suggested a significant association between oral diseases and an increased incidence and risk of vascular disease (5).

Multiple prospective cohorts and meta-analyses indicate that even after controlling for age, smoking, blood pressure, lipids, and glycemic abnormalities, periodontal disease remains significantly associated with coronary heart disease (CHD), stroke, and cardiovascular mortality. Pooled hazard ratios (HRs) are generally in the range of 1.2–1.4, which are modest at the individual level but potentially meaningful at the population level given the high prevalence of periodontitis in adults. For context, the population attributable fraction (PAF)—the proportion of events that would be preventable if the association were causal—can be expressed as PAF = Pe(RR−1)/[1+Pe(RR−1)], where Pe is exposure prevalence (6). Using commonly cited prevalence estimates of ~45–50% (7), an RR/HR of 1.2–1.4 corresponds to a hypothetical PAF of roughly ~8–17%; however, because PAF is sensitive to confounding control and exposure definition, these values should be interpreted as illustrative bounds rather than causal attribution. Given residual confounding and study heterogeneity, the nature of this relationship still requires more rigorous study designs (5, 8).

Based on these observations and clinical experience, this article adopts the integrative framework of the oral–vascular axis and aims to delineate how the oral ecosystem, particularly periodontal tissues and their microbiota, may constitute a persistent source of systemic inflammatory and immune signals relevant to atherosclerotic cardiovascular disease (ASCVD). Numerous reviews and consensus statements have already synthesized the epidemiologic association between periodontitis and ASCVD. They emphasize that the available evidence is dominated by observational cohorts and surrogate-endpoint intervention studies, which support association and biological plausibility but do not, by themselves, establish a cause–effect relationship or demonstrate event-level cardiovascular benefit. Against this backdrop, an ongoing challenge for narrative synthesis is that the underlying evidence spans heterogeneous biological scales, including biofilm ecology, barrier biology, innate and adaptive immunity, immunometabolic remodeling, and vascular wall pathophysiology, as well as disparate evidentiary tiers. Consequently, many syntheses, often appropriately given scope constraints, present candidate mechanisms in parallel, such as transient bacteremia, systemic inflammatory spillover, adaptive immune circuits, and metabolite-mediated pathways. This structure can make it difficult to follow how processes interact across compartments and time, or to keep inferential boundaries explicit when moving from mechanistic feasibility in *in vitro* and animal systems to human plausibility and, separately, to clinical benefit (7, 9, 10).

A related integrative gap is that, despite rapid advances in oral microbiome science, functional oral microecology is not always foregrounded as an explanatory layer that can tune systemic immune activation thresholds and help explain heterogeneity in vascular responses. Increasingly, broader oral–systemic frameworks emphasize functional outputs as candidate mediators rather than ancillary descriptors of dysbiosis (11, 12). In parallel, longitudinal multi-omics studies demonstrate coordinated relationships among plaque microbial functions, local metabolite states, and host inflammatory readouts across gingival health–gingivitis transitions and in periodontitis with treatment response. However, integrated human designs that simultaneously resolve oral functional states, host immunophenotypes, and within-person vascular phenotypes over time remain comparatively limited, constraining the extent to which effect modification and stratified pathways can be synthesized with high confidence from the current literature (13, 14). To address these integrative challenges, the present review offers an immunologically oriented synthesis structured around a spatiotemporal dual-regulatory network of immune and metabolic processes. This framework emphasizes how variation in oral microecology and immune phenotypes may stratify vascular susceptibility, while maintaining a consistent distinction between association, mechanistic plausibility, experimental feasibility, and causal inference (2).

## 2 Clinical and epidemiological associations

The oral–vascular axis refers to a multidirectional and dynamic relationship between the oral ecosystem, particularly periodontal tissues and microbiota, and the vascular system. CVD is highly prevalent and burdensome, whereas periodontitis is common, long-standing, and characterized by chronic inflammation. Population-based studies generally report a positive association between periodontitis and multiple cardiovascular outcomes that often persists after adjustment for traditional risk factors, although residual confounding remains plausible. This chapter summarizes population-level associations, graded relationships between periodontitis severity and cardiovascular risk, and intervention evidence based on functional and structural vascular surrogate endpoints, thereby providing magnitude references and boundary conditions for mechanistic interpretation.

### 2.1 Overall association between periodontal disease and CVD

Across regions, prospective cohort studies and meta-analyses consistently show a positive association between periodontal disease and CVD. A meta-analysis of 39 prospective cohorts including 4,389,263 participants reported that periodontal disease was associated with a relative risk of 1.24 (95% confidence interval [CI] 1.15–1.34) for major adverse cardiovascular events (MACE), with consistent associations for (CHD) (1.20), myocardial infarction (MI) (1.14), stroke (1.26), and cardiovascular death (1.42); all-cause mortality was also elevated (1.31) (8).

An umbrella review with a higher level of evidence re-evaluated existing systematic reviews and meta-analyses and confirmed the overall association, while highlighting uncertainty arising from heterogeneity in exposure and outcome definitions, confounding control, and population differences. Current evidence is insufficient to support a strong causal claim and is best interpreted as a consistent, directionally aligned association at the population level. Whether any component of this association is causal remains uncertain and will require triangulation across causal-inference designs, mechanistic evidence, and adequately powered event-driven trials (5, 8). To illustrate how these overall associations appear across different populations and study designs, a selection of key observational cohort studies is summarized in **Table 1**. Because the included studies are observational, the reported findings reflect associations and do not allow causal inference.

**Table 1:** Selected observational studies on periodontitis and cardiovascular outcomes.

Study (first author, year)	Design and population	Periodontitis exposure	Cardiovascular outcomes	Effect size (HR/OR/RR/IRR with 95% CI)	Main findings
Hansen 2016 (124)	Danish nationwide registry; 17,691 patients with hospital-diagnosed periodontitis and matched controls	Hospital discharge diagnosis of periodontitis (severe cases)	Myocardial infarction, ischemic stroke, cardiovascular death, major adverse cardiovascular events, all-cause mortality	Cardiovascular death: IRR 2.02 (1.87–2.18); All-cause mortality: IRR 2.70 (2.60–2.81).	Severe periodontitis was associated with about 2-fold higher cardiovascular mortality and nearly 3-fold higher all-cause mortality compared with controls, with likely residual confounding.
Wallin Bengtsson 2021 (125)	Swedish community-based cohort, age ≥60 years; 858 patients, 17-year follow-up	Radiographic bone loss used to define periodontitis severity	Incident ischaemic heart disease, stroke, all-cause mortality	Ischemic heart disease incidence (all participants): HR 1.5 (1.1–2.1). All-cause mortality (all participants): HR 1.4 (1.2–1.8).	Periodontitis was associated with higher risks of ischaemic heart disease and all-cause mortality (especially in women), but showed no significant association with stroke.
Chen 2024 (126)	NHANES; adults with established cardiovascular disease, 2,135 patients with CVD	CDC/AAP categories of periodontitis (none or mild, moderate, severe)	All-cause and cardiovascular mortality among patients with cardiovascular disease	All-cause mortality: HR 1.25 (1.02–1.52) for moderate/severe vs no/mild. Severe CAL: HR 1.07 (1.01–1.14)	Among adults with established cardiovascular disease, moderate-to-severe periodontitis was associated with higher all-cause mortality, but not with cardiovascular- or cancer-specific mortality.
Guo 2025 (127)	NHANES; 9,202 adults free of clinical cardiovascular disease at baseline; 17.5-year follow-up	Periodontitis graded as none or mild versus moderate-to-severe	High-sensitivity troponin, NT-proBNP, cardiovascular mortality	Cardiovascular mortality (moderate–severe vs no periodontitis): HR 1.449 (1.027–2.044)	Periodontitis was associated with higher cardiac biomarker levels and increased cardiovascular mortality, while single baseline measurements and unmeasured factors may still confound the association.
Reichert 2024 (128)	Prospective cohort of 1,002 patients with angiographically proven cardiovascular disease; 10-year follow-up	Severe periodontitis defined as ≥30% of teeth with proximal CAL ≥5 mm	Recurrent myocardial infarction, stroke or transient ischaemic attack, cardiovascular death	Combined endpoint: adjusted HR 1.26 (1.0–1.58) for severe periodontitis	Severe periodontitis was associated with a higher 10-year risk of a composite endpoint including recurrent myocardial infarction, stroke or transient ischaemic attack, and cardiovascular death.

### 2.2 Graded association between periodontitis severity and cardiovascular risk

Compared with tooth loss, which reflects cumulative damage, using clinically defined periodontitis severity and extent as exposure more closely captures the dynamic nature of the oral–vascular axis. Recent prospective and long-term follow-up studies in various populations demonstrate that with increasing severity and extent of periodontitis, the risk of adverse cardiovascular outcomes and mortality rises in a graded fashion. After multivariable adjustment, the direction of association is preserved, though effect sizes vary depending on definitions and measurement methods.

In high-risk populations, a hypertensive subgroup from National Health and Nutrition Examination Survey (NHANES) (n=5,665; mean follow-up ~10.2 years) stratified participants based on clinical periodontal examination. Compared with individuals without periodontitis, those with periodontitis had higher risks of cardiovascular mortality (HR 1.48, 95% CI 1.15–1.89) and all-cause mortality (HR 1.33, 95% CI 1.18–1.51), with a trend of increasing risk across severity categories. Multivariable models and sensitivity and subgroup analyses showed stable directions, consistent with the possibility that, in high cardiometabolic risk settings, periodontal inflammatory burden may more readily track with adverse vascular phenotypes and outcomes (15).

Using continuous measures, the Study of Health in Pomerania (SHIP)-START cohort (mean follow-up ~13 years) quantified periodontal exposure with mean probing pocket depth (PPD), clinical attachment loss (CAL), and composite periodontal scores. Each standard-deviation increase in these measures was associated with higher all-cause and cardiovascular mortality. Importantly, there was a significant additive interaction between periodontal burden and systemic low-grade inflammation, accounting for a component of excess cardiovascular mortality in interaction models, while also supporting biological plausibility (16).

From an incident event perspective, the Atherosclerosis Risk in Communities Study (ARIC) study mapped full-mouth clinical periodontal examinations to Centers for Disease Control and Prevention/American Academy of Periodontology (CDC/AAP) and periodontal profile class (PPC) classification systems and evaluated incident (CHD) using competing-risk models. More severe and extensive periodontal phenotypes were positively associated with CHD events, with consistent gradients from mild to severe and localized to generalized disease, despite variation in effect sizes across classification schemes. Differences in full-mouth versus partial-mouth assessments, metric selection, and staging/classification systems limit comparability of effect estimates and contribute to heterogeneity (17).

Integrative evidence from umbrella reviews similarly highlights that when exposure is defined by clinical periodontal staging and extent rather than self-report or tooth loss, associations with CVD outcomes are more stable and interpretable. Nevertheless, residual confounding and reverse causality cannot be fully excluded. Thus, this gradient relationship is best viewed as a directionally coherent pattern of associations providing population-level constraints and magnitude references for mechanistic work, rather than proof of causality (5).

Reverse causality and bidirectionality should also be considered when interpreting severity gradients between periodontitis and ASCVD. ASCVD and its cardiometabolic correlates are accompanied by chronic low-grade inflammation, endothelial dysfunction, and immune dysregulation, and they share major behavioral and socioeconomic determinants with periodontitis (e.g., smoking, diabetes, obesity, and access-to-care). As a result, a graded association may be compatible not only with oral-to-vascular mechanistic hypotheses but also with shared susceptibility and multimorbidity clustering, which can inflate apparent dose–response patterns even under extensive covariate adjustment. Consistent with this interpretation, an updated American Heart Association scientific statement emphasizes that current evidence supports an association but does not, by itself, establish a cause–effect relationship between periodontitis and ASCVD (10). In addition, a large nationwide linked-cohort analysis in Korea reported reciprocal longitudinal associations, with periodontitis associated with subsequent CVD risk and baseline CVD also associated with subsequent periodontitis risk after multivariable adjustment; these findings are compatible with bidirectionality, but they do not resolve residual confounding or shared-cause structures (18). Finally, causal-inference approaches that are less vulnerable to confounding and reverse causation have generally not provided strong support for a causal effect of periodontitis on coronary artery disease or stroke, reinforcing the need to interpret observational gradients as evidence of correlation and biological plausibility rather than definitive causal direction (19).

### 2.3 Periodontal therapy and cardiovascular outcomes

Regarding intermediate mechanistic endpoints, randomized controlled trials (RCTs) and meta-analyses generally report short- to medium-term improvements in endothelial-function surrogates and reductions in systemic inflammatory markers after non-surgical periodontal therapy (NSPT), although effect sizes vary, and between-study heterogeneity is substantial. A recent meta-analysis showed that after NSPT, flow-mediated dilation (FMD) increased and C-reactive protein (CRP) and interleukin-6 (IL-6) decreased. When follow-up was ≤3 months, FMD improvement was approximately 1–4%; when follow-up was ≥6 months, the effect attenuated, suggesting that durability may be influenced by supportive periodontal therapy schedules, adherence, and the timing/intensity of the initial intervention (20).

Other reviews similarly report that NSPT exerts consistent but variably sized beneficial effects on FMD, carotid intima–media thickness (cIMT), and some arterial stiffness indices, again with considerable between-study heterogeneity (21).

Interpretation of surrogate-endpoint improvements after periodontal therapy requires caution, because both the magnitude and inferential meaning of changes in FMD/cIMT are constrained by measurement variability, protocol heterogeneity, and background cardiometabolic management. Prognostic meta-analyses indicate that lower baseline brachial FMD is associated with higher future cardiovascular event risk (e.g., pooled risk estimates on the order of ~0.92 per 1% higher FMD), supporting its relevance as a vascular health marker; however, this does not imply that a short-term, post-intervention increase of ~1–4 percentage points necessarily translates into durable vascular remodeling or reduced ASCVD events (22). Within-individual variability and technical reproducibility further complicate inference: FMD reliability improves substantially when contemporary consensus protocols are followed, but measurement error remains substantial and depends on adherence to guideline-recommended acquisition and analysis procedures, so modest absolute changes may be difficult to distinguish from variability unless assessments are repeated and standardized across equipment, analytic pipelines, operators, cuff placement, and timing relative to circadian state, meals, and medications (23, 24). Therefore, modest absolute improvements, particularly near the lower end of the reported range, may not consistently exceed within-subject test–retest variability unless measurements are repeated and standardized. Differences in maintenance regimens and their reporting can affect periodontal stability and systemic inflammatory reduction, contributing to inconsistent vascular readouts over follow-up. In addition, periodontal intervention trials are heterogeneous with respect to baseline disease severity, treatment intensity, adjunctive antimicrobials or extractions, and whether supportive maintenance is implemented and clearly described, all of which can attenuate or amplify systemic inflammatory changes and contribute to between-study inconsistency in vascular outcomes (10, 20). Finally, concomitant cardiovascular therapies (notably statins and antihypertensive agents) can independently improve endothelial function as captured by FMD (25, 26), and changes in medication initiation, dose, or adherence during follow-up can confound attribution of modest FMD gains to periodontal treatment alone. Accordingly, the existing intervention literature should be framed as supporting biological plausibility and experimental feasibility for an oral–vascular link at the level of intermediate phenotypes, while event-level cardiovascular benefit remains unproven and requires adequately powered, event-driven trials with rigorous control of concurrent therapies and harmonized vascular phenotyping.

For structural endpoints, a randomized 2-year trial (NCT03072342) provided prospective evidence: compared with usual care, intensive periodontal therapy (IPT) slowed cIMT progression and produced early improvements in FMD and reductions in inflammatory and oxidative stress markers. The trial further used evidence-based surrogacy data to approximate potential cardiovascular benefit, but given ongoing debates about the strength of extrapolation from cIMT to future events, interpretation must consider population characteristics and intervention context (27, 28).

Regarding hard clinical endpoints such as MI, stroke, and CVD death, a Cochrane review and recent secondary analyses conclude that current RCT evidence is insufficient to assert that periodontal treatment reduces clinical events. Limitations include small sample sizes, short follow-up, heterogeneous patient selection, differences in intervention intensity and maintenance strategies, and potential dilution by optimized cardiovascular care (29).

In summary, population data show that periodontal burden is directionally aligned and quantitatively associated with adverse cardiovascular phenotypes and outcomes. Periodontal treatment can improve endothelial function and inflammatory profiles in the short to medium term and may slow arterial remodeling. Explaining this linkage requires scrutiny of the underlying biological pathways of the oral–vascular axis—from local dysbiosis and barrier disruption, through inflammatory spill-over and immune amplification, to reversible endothelial dysfunction and plaque formation—with particular attention to the amplifying roles of immune cross-reactivity and microbial metabolites.

## 3 Core immunological mechanisms: pathways from local infection to systemic vascular disease

Chronic periodontitis has been repeatedly associated with ASCVD, but the biological linkage is more plausibly explained by interlocking, multi-scale pathways than by a single linear mechanism. Within the oral–vascular axis framework, the relevant exposure extends beyond microbial presence to include functional oral microecology, such as barrier-disruptive activity, immunostimulatory ligands, vesicle-mediated cargo delivery (including outer membrane vesicles [OMVs]), and metabolic outputs, which engage host defenses locally and may influence systemic inflammatory set-points. These elements can be conceptualized as a dual-regulatory network of immune and metabolic processes in which microbial signals and host immunometabolic programs jointly condition vascular susceptibility.

Systemic exposure arises through episodic dissemination of whole bacteria and through more sustained trafficking of microbial products and particulate cargo (e.g., OMVs and pathogen-associated molecular patterns [PAMPs]/damage-associated molecular patterns [DAMPs]). These signals are sensed by endothelial and myeloid pattern-recognition systems and are integrated with circulating inflammatory mediators, acute-phase responses, and lipoprotein remodeling, thereby shaping a permissive milieu for vascular activation. In parallel, immune-mediated amplification can arise from durable shifts in hematopoiesis and innate immune responsiveness consistent with trained immunity, as well as from adaptive immune circuits involving T-cell polarization and cross-reactive antibodies and immune-complex effector pathways. Across these interacting processes, endothelial activation with reduced nitric oxide (NO) bioavailability represents a plausible convergence phenotype that links systemic immune tone to leukocyte recruitment, plaque progression, and thromboinflammatory complications.

**Figure 1:**
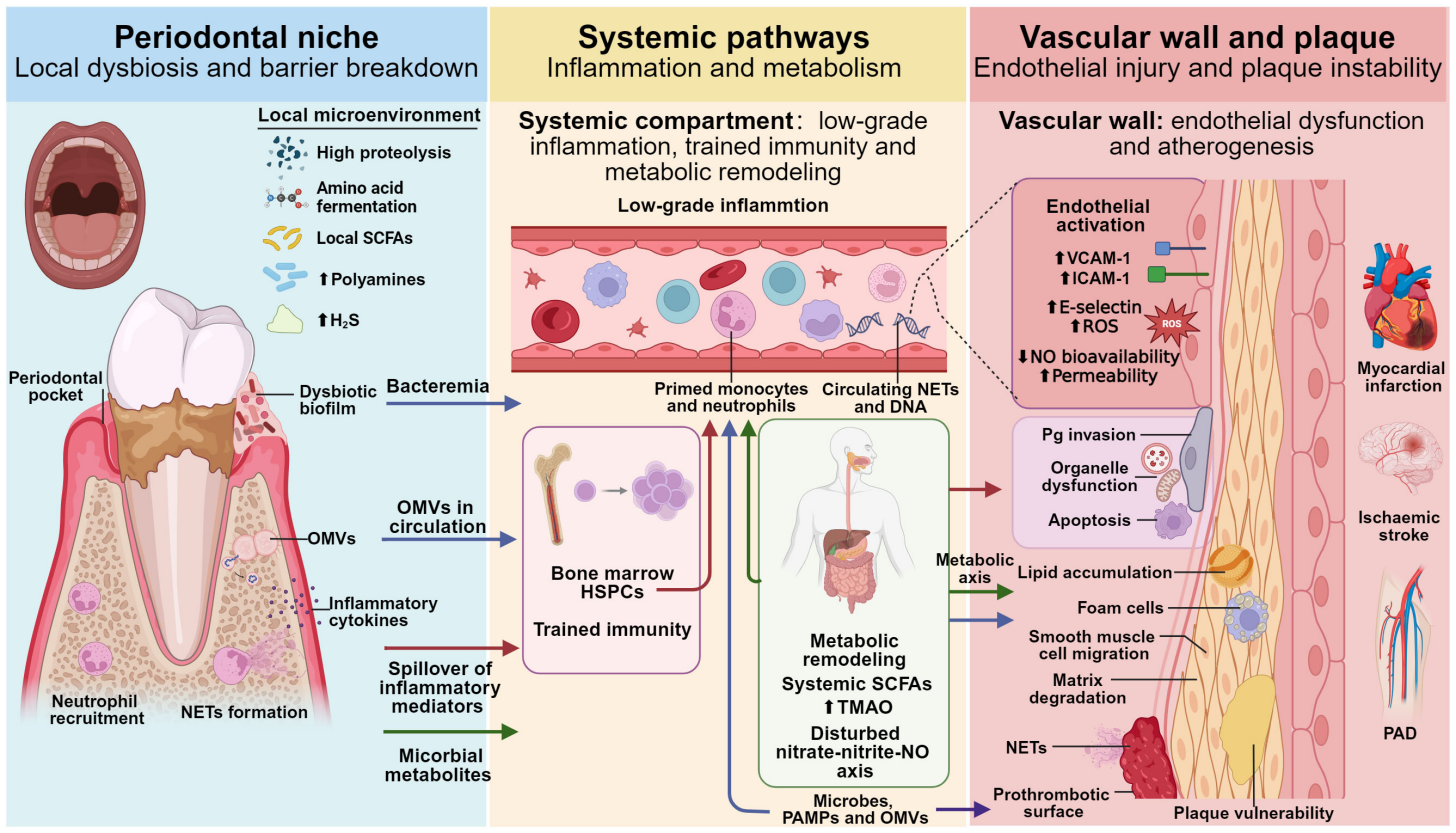
Overview of the oral–vascular axis linking chronic periodontitis to ASCVD.The left panel shows the periodontal niche, where dysbiotic biofilms, metabolic shifts, epithelial barrier disruption and NET formation lead to the release of bacteria, OMVs, inflammatory mediators and microbial metabolites. The central panel depicts systemic pathways, including low-grade inflammation, trained immunity in the bone marrow and metabolic remodelling (systemic SCFAs, TMAO and nitrate–nitrite–NO axis). The right panel illustrates vascular wall and plaque changes, with endothelial activation and dysfunction, direct effects of periodontal pathogens and OMVs, lipid accumulation, foam-cell formation, matrix degradation, plaque vulnerability and NET-related thrombosis; coloured arrows indicate inflammatory and trained-immunity (red), metabolic (green), microbial (blue) and NET–thrombosis (purple) pathways. Created with BioRender.

Evidence supporting these steps is heterogeneous. Experimental systems frequently establish mechanistic feasibility and, in some contexts, directionality, whereas human observational cohorts, plaque and ex vivo analyses, and surrogate-endpoint intervention studies more commonly support association and biological plausibility than proven causality. Accordingly, Sections 3.1–3.5 progress from local dysbiosis and barrier disruption, to systemic propagation and immunometabolic remodeling, to endothelial dysfunction and lesion evolution, and then to modifiers that may contribute to inter-individual heterogeneity, including antigen-specific adaptive immune circuits and microbe–host metabolites that modulate redox balance and NO homeostasis. **Figure 1** provides a conceptual overview of these interconnected mechanisms.

### 3.1 Local dysbiosis and inflammatory focus

A healthy periodontal niche is maintained by a diverse microbiota, an intact epithelial and connective-tissue barrier, and a low-grade activated mucosal immune system (30, 31). As dental plaque matures and the microenvironment shifts (reduced oxygen tension, altered potential of hydrogen [pH] and redox potential, and increased availability of blood-derived nutrients), the community can transition from symbiosis to dysbiosis. In the oral–vascular axis framework, the relevant biological “exposure” is therefore not only taxonomic composition but functional oral microecology that interfaces with host defenses and shapes downstream inflammatory set-points through barrier-disruptive activities, immunostimulatory ligands, vesicle-mediated cargo transfer, and local metabolic outputs. Under these conditions, inflammation-prone taxa such as *Porphyromonas gingivalis, Treponema denticola, and Tannerella forsythia* may gain a relative advantage, and local inflammation tends to persist (32–34).

Barrier disruption is an early and critical event that increases trans-barrier flux of microbial and host-derived signals. OMVs and virulence factors such as lipopolysaccharide (LPS), fimbriae, and proteases (gingipains) can displace or down-regulate tight-junction proteins (e.g., zonula occludens 1 [ZO-1] and occludin) and reduce transepithelial electrical resistance, thereby increasing permeability. Comparable barrier phenotypes have been demonstrated in endothelial models, supporting mechanistic parallels between mucosal and vascular interfaces (35, 36).

Once barrier integrity is compromised, innate immune activation becomes self-reinforcing. Pathogen-associated and damage-associated molecular patterns (PAMPs/DAMPs; microbial motifs and host-derived danger signals released during tissue injury) signal through Toll-like receptors 2/4 (TLR2/4) and nucleotide-binding oligomerization domain (NOD) receptors, activating myeloid differentiation primary response 88 (MyD88)–nuclear factor κB (NF-κB) and mitogen-activated protein kinase (MAPK) pathways that up-regulate chemokines and adhesion programs and promote recruitment and activation of monocytes and neutrophils. Complement–TLR cross-talk further modulates inflammatory thresholds: *P. gingivalis* gingipains can engage the complement component 5a (C5a) axis and alter phagocytic set-points, and inhibition of C5a–complement component 5a receptor (C5aR) signaling in animal and local intervention studies attenuates inflammation and bone loss (37). In parallel, NLR family pyrin domain containing 3 (NLRP3) inflammasome assembly promotes maturation of interleukin-1β (IL-1β) and interleukin-18 (IL-18), linking innate sensing to matrix degradation and bone resorption phenotypes (38, 39). Collectively, these pathways are well supported mechanistically in cellular and animal models, whereas human data more strongly support association and biological plausibility than directional causality.

As biofilms mature, community-level functional outputs further strengthen chronicity. Quorum-sensing supports coordinated division of labor (40), and OMVs act as inter-kingdom cargo carriers delivering LPS, proteases, and small ribonucleic acids (RNAs), thereby reshaping host and microbial pathways across cell types (41). OMVs can be internalized by epithelial cells and may evade complete degradation, prolonging exposure to immunostimulatory signals and helping to sustain inflammation even at relatively low bacterial loads (35, 42). In parallel, local immunometabolic remodeling accompanies dysbiosis, including enhanced proteolysis and amino-acid fermentation, altered short-chain fatty acid (SCFA) composition, and increased sulfur metabolism and polyamine shifts; over days to weeks, these changes can lower activation thresholds in epithelial and innate immune cells and sensitize responses to subsequent stimuli (43, 44). When neutrophil extracellular trap (NET) formation exceeds clearance capacity, NETs amplify matrix damage and tissue injury and correlate positively with clinical severity (45), facilitating pocket deepening and reinforcing chronic inflammatory signaling (31).

Systemic exposure can arise even at this local stage through two conceptually distinct routes that are separated in later sections: episodic dissemination of whole bacteria (transient bacteremia) and more sustained leakage of particulate or soluble inflammatory signals (OMVs and PAMPs/DAMPs). Everyday activities such as tooth brushing, flossing, chewing, and routine dental procedures may induce transient, low-grade bacteremia that is rapidly cleared in most individuals but could exert cumulative effects in susceptible hosts (46, 47). In contrast, the systemic access of OMVs and soluble ligands may provide a more persistent background favoring endothelial and myeloid activation (30, 48). At the endothelial level, such inflammatory signaling has been linked in experimental systems to reduced endothelial nitric oxide synthase (eNOS) activity, increased reactive oxygen species, and up-regulation of intercellular adhesion molecule 1 (ICAM-1), vascular cell adhesion molecule 1 (VCAM-1), and selectins, thereby promoting monocyte adhesion and transendothelial migration; vascular smooth muscle cells (SMCs) may undergo a shift toward a synthetic phenotype (49–51). The extent to which these local processes translate into sustained systemic inflammatory tone is likely to vary across individuals, reflecting differences in barrier integrity, neutrophil-dominant inflammatory programs (including NET propensity), and resolution capacity at the periodontal interface. Collectively, these processes can sustain a chronic inflammatory lesion characterized by periodontal pocket formation and progressive tissue destruction, while increasing the probability of systemic inflammatory spill-over (30, 38).

### 3.2 Systemic propagation and immunometabolic remodeling

Once mucosal permeability is increased and local inflammatory outputs intensify, periodontal lesions can become a sustained source of systemic immune and metabolic perturbation. At the mechanistic level, systemic propagation reflects the combined effects of episodic dissemination of whole bacteria (transient bacteremia) and more persistent circulation of microbial products and vesicular cargo (e.g., OMVs, LPS, and protein/lipid fragments), which are subsequently integrated and amplified by the liver, bone marrow, and vascular wall into a low-grade pro-inflammatory state.

Bacteremia typically resolves within minutes to hours and involves low bacterial loads that most hosts clear efficiently; however, in individuals with valvular disease, vascular implants, or pre-existing vascular lesions, repeated low-dose exposures may have cumulative effects (31, 52). In parallel, particulate and soluble microbial signals may persist longer than intact bacteria and can be less readily eliminated, potentially increasing opportunities for downstream sensing within blood and vascular compartments. Consistent with systemic trafficking of oral microbial material, multiple studies have detected deoxyribonucleic acid (DNA) of oral pathogens and associated virulence-factor signals in human atherosclerotic plaques and large-vessel specimens (53, 54). These findings support biological plausibility for vascular exposure to oral-derived microbial components, while interpretation should remain cautious with respect to viability and the extent of direct colonization.

Spill-over of inflammatory mediators provides a bridge between oral inflammation and systemic immunometabolic remodeling. Cytokines released from periodontal tissues (including IL-1β, IL-6, tumor necrosis factor-α [TNF-α], and monocyte chemoattractant protein 1 [MCP-1]) enter the circulation and can engage hepatic acute-phase responses, increasing CRP, serum amyloid A (SAA), fibrinogen, and complement components. In parallel, high-density lipoprotein (HDL) function is impaired: apolipoprotein A-I (ApoA-I) is displaced by SAA, and activities of lecithin–cholesterol acyltransferase (LCAT) and paraoxonase decline. This constellation can weaken cholesterol efflux and antioxidant capacity while shifting the circulating milieu toward a more pro-inflammatory and prothrombotic state (48). Functionally, such lipoprotein remodeling provides a metabolic context in which inflammatory signaling and oxidant stress may be more readily sustained, thereby coupling systemic metabolism to immune activation thresholds.

At the vascular wall, repeated low-dose exposure to cytokines and inflammatory ligands can promote an alert but still partially functional endothelial “pre-activation” phenotype characterized by reduced eNOS activity and NO bioavailability, increased nicotinamide adenine dinucleotide phosphate (NADPH) oxidase–derived reactive oxygen species, and up-regulation of ICAM-1/VCAM-1 and selectins, thereby facilitating monocyte adhesion and transmigration. Importantly, clinical studies using surrogate endpoints report that standard periodontal treatment reduces IL-6 and CRP and improves FMD in parallel, consistent with partial reversibility of endothelial dysfunction at the level of surrogate physiology—while still not constituting proof of causal event reduction (20, 55).

Systemic amplification may also involve trained immunity, defined as durable epigenetic and metabolic reprogramming of innate immune cells and/or progenitors that enhances responsiveness to secondary stimulation. In monocytes and macrophages, this phenotype has been associated with increased histone H3 lysine 4 trimethylation (H3K4me3)/histone H3 lysine 27 acetylation (H3K27ac) and up-regulation of glycolysis, cholesterol biosynthesis, and glutaminolysis, with consequent augmentation of IL-1β/IL-6 responses and oxidative burst (38, 39, 56). At the bone marrow level, myeloid-biased hematopoiesis and expansion of pro-inflammatory cell pools may emerge, plausibly lowering vascular activation thresholds and making endothelial adhesion and transmigration more readily triggered by metabolic or hemodynamic fluctuations. In this framework, immunometabolic remodeling is not simply a correlate of inflammation but a potential mechanism by which inflammatory exposures are “remembered” within innate immune compartments over longer time scales.

Through these interconnected processes: recurrent bacteremia and persistence of microbial cargo, cytokine spill-over with acute-phase and lipoprotein remodeling, and trained immunity with hematopoietic bias, intermittent local inflammation can be amplified into a sustained systemic pro-inflammatory state that aligns with observed changes in vascular function (FMD, pulse-wave velocity) and inflammatory markers (high-sensitivity C-reactive protein [hs-CRP], IL-6) (57). The magnitude and durability of this systemic imprint are likely to vary across individuals, reflecting differences in baseline cardiometabolic context, hepatic acute-phase responsiveness, and myeloid inflammatory set-points, and may therefore contribute to heterogeneity in downstream vascular phenotypes. This systemic milieu provides the mechanistic backdrop for the endothelial dysfunction and lesion evolution discussed next.

### 3.3 Endothelial dysfunction and plaque evolution

Within the systemic milieu described above—characterized by low-grade inflammatory spillover, acute-phase and lipoprotein remodeling, and myeloid priming consistent with trained immunity—endothelial dysfunction represents an early, quantifiable vascular phenotype that can integrate immune and metabolic inputs. In this context, reduced nitric oxide (NO) bioavailability and impaired endothelium-dependent vasodilation are mechanistically linked to cytokine-driven inhibition or uncoupling of eNOS and to amplified oxidative stress. Pro-inflammatory cytokines such as TNF-α and IL-1β inhibit or uncouple eNOS and increase reactive oxygen species via NADPH oxidase, thereby attenuating NO-mediated vasodilation and diminishing NO’s anti-adhesive, anti-inflammatory, and antithrombotic functions (49, 50). Reduced NO bioavailability therefore provides a plausible convergence phenotype connecting systemic immune tone to endothelial activation and leukocyte recruitment, while remaining distinct from evidence for causal event reduction.

Beyond systemic immune and metabolic drivers, oral microbes and their products may also influence the endothelial microenvironment. *In vitro* and animal studies demonstrate mechanistic feasibility for *P. gingivalis* adherence to, and invasion of, vascular cells, with perturbations in adhesion molecule expression and cellular homeostasis (58). In human vascular specimens, signals consistent with oral microbial exposure (e.g., bacterial DNA and virulence-factor signatures) are more commonly detected than viable organisms, supporting biological plausibility of trafficking of microbial material while underscoring that inferences about viability or sustained colonization require caution (59–61). Thus, available human data are generally more consistent with vascular exposure to microbial components than with stable colonization as a necessary prerequisite for lesion biology.

Once the endothelium is activated, leukocyte recruitment becomes more permissive. Activated endothelial cells up-regulate VCAM-1, ICAM-1, and selectins, and local chemokines support monocyte rolling, firm adhesion, and transendothelial migration into the intima. There, in a milieu shaped by oxidized lipoproteins and inflammatory cues, monocytes differentiate into macrophages and acquire lipids to form foam cells, establishing the cellular basis for early plaque formation (62, 63). Importantly, this sequence does not require direct vascular infection; rather, it is compatible with a “lowered activation threshold” state in which systemic immunometabolic perturbations amplify vascular responsiveness to common atherogenic stimuli.

During lesion progression, SMCs undergo phenotypic switching from a contractile to a synthetic-inflammatory state; subsets adopt macrophage-like features and contribute to extracellular matrix turnover and fibrous-cap remodeling. This shift can promote imbalance in collagen metabolism and cap thinning, particularly in the setting of persistent inflammation and lipid loading. In parallel, persistent inflammatory tone can promote matrix degradation programs and impair repair responses, contributing to structural vulnerability.

A prothrombotic milieu further contributes to plaque complications. Up-regulation of tissue factor, platelet activation, and NET formation promote reciprocal amplification of coagulation and inflammation, increasing susceptibility to plaque erosion or rupture. Imaging and pathology studies indicate distinct morphological features and triggers for erosion versus rupture, but both are consistently linked to heightened inflammatory burden and endothelial dysfunction (64–66).

Together, these mechanisms connect systemic inflammatory and immunometabolic priming to vascular wall phenotypes through endothelial dysfunction, and then to plaque initiation, structural vulnerability, and downstream clinical events. Because endothelial dysfunction is relatively reversible and quantifiable, measures such as FMD, soluble ICAM-1/VCAM-1, and inflammatory markers can serve as useful surrogate readouts for mechanistic studies and early-phase trials, while remaining distinct from evidence for clinical event reduction (57). Sections 3.4 and 3.5 then address how antigen-specific adaptive immune circuits and microbe–host metabolites can further modulate this primed vascular state and contribute to inter-individual heterogeneity.

### 3.4 Immune cross-reactivity and adaptive immune circuits

Inter-individual heterogeneity in vascular phenotypes despite comparable microbial exposures suggests that host immunophenotype and antigen-specific immune responses modulate the vascular consequences of periodontal inflammation. A mechanistic hypothesis is that cross-reactive humoral immunity and immune-complex effector pathways amplify endothelial activation beyond that attributable to nonspecific systemic cytokine exposure alone.

Protein citrullination (post-translational conversion of peptidyl-arginine to citrulline) generates neo-epitopes that can breach tolerance and promote anti-citrullinated protein antibody (ACPA) responses. In experimental systems, Porphyromonas gingivalis peptidylarginine deiminase (PPAD) can increase the burden of citrullinated epitopes, while *Aggregatibacter actinomycetemcomitans* leukotoxin (LtxA) induces neutrophil hypercitrullination, potentially broadening the autoantigen repertoire (67). These mechanisms are most strongly supported in rheumatoid arthritis–associated autoimmunity; extrapolation to cardiovascular phenotypes outside RA should therefore be framed as mechanistic plausibility rather than an established causal linkage (68).

Once ACPAs are generated, a coherent downstream sequence is (neo-epitope formation → break in tolerance → ACPA generation → immune-complex formation → complement/Fc gamma receptor [FcγR] effector activation), which can amplify vascular inflammation beyond nonspecific systemic cytokine tone. Cross-reactive antibodies can bind citrullinated or structurally similar epitopes and form immune complexes that activate complement and Fcγ receptor pathways on endothelial and myeloid cells. Complement–TLR cross-talk, including the C5a–C5aR axis, can further lower inflammatory thresholds, and these convergent pathways can increase reactive oxygen species, up-regulate ICAM-1/VCAM-1 and chemokines, and impair eNOS/NO signaling—features consistent with an endothelial “pre-activation” phenotype described in the preceding sections (37, 49, 50). Importantly, this conceptual framework does not require direct vascular infection; rather, it posits that antigen-specific effector responses can magnify vascular activation in the presence of a priming systemic inflammatory milieu.

Cellular immunity likely contributes in parallel, particularly in sustaining chronicity and shaping macrophage phenotypes. Persistent antigen exposure within periodontal lesions can promote T-cell polarization and dysregulated effector–regulatory balance, which may reinforce myeloid activation and endothelial inflammation within the vascular wall. In this context, immunoregulatory checkpoint pathways (e.g., programmed cell death protein 1 (PD-1)/programmed death-ligand 1 (PD-L1) and cytotoxic T-lymphocyte–associated protein 4 (CTLA-4)) represent key restraints on T-cell activation that help maintain chronic inflammatory set-points; altered checkpoint restraint, compensatory exhaustion programs, or inadequate regulatory buffering could plausibly modulate the intensity and persistence of downstream vascular inflammation in susceptible hosts. While checkpoint biology is well established in chronic inflammatory contexts, direct periodontal-to-vascular checkpoint mechanisms remain less well resolved and should be presented as testable hypotheses rather than proven links.

Within plaques, autoimmunity and thromboinflammation can reinforce each other. NETs expose citrullinated histones and cytoskeletal components, providing abundant potential autoantigen sources that can support epitope spreading, while tissue factor–platelet interactions increase thrombogenicity and promote leukocyte recruitment, linking immune effector activity to plaque vulnerability (64). Consistent with this concept, reviews of vulnerable plaque biology emphasize that adaptive and innate immune responses jointly shape inflammatory intensity and remodeling of the fibrous cap, thereby influencing susceptibility to erosion or rupture (66).

At the population level, ACPAs are consistently associated with heightened cardiovascular risk in rheumatoid arthritis, whereas results are more heterogeneous in non-RA populations; larger samples with standardized antigen panels are needed to clarify independent contributions beyond shared inflammation and cardiometabolic comorbidity (69). Overall, periodontal pathogen-related enzymes and leukotoxins can increase the burden of neo-epitopes and hypercitrullination, promoting cross-reactive antibodies and immune complexes that—via complement and Fc receptor pathways—may amplify endothelial activation and prothrombotic tendencies, superimposing on the reversible endothelial dysfunction described above and contributing to inter-individual heterogeneity in vascular outcomes.

### 3.5 Microbial metabolites and vascular modulation

Microbe-derived metabolites represent a functional interface through which oral microecology may influence systemic immunometabolic state and vascular physiology. Candidate metabolite classes implicated in endothelial function, inflammatory tone, and thromboinflammatory balance converge on redox regulation and NO bioavailability. Across metabolite classes, mechanistic directionality is most often supported by experimental studies, whereas human evidence more commonly supports association and biological plausibility rather than proven causality.

SCFAs, including acetate, propionate, and butyrate, can modulate immune and vascular pathways through G-protein–coupled receptors (e.g., G protein–coupled receptor 41 [GPR41]/G protein–coupled receptor 43 [GPR43]) and epigenetic regulation via histone deacetylase inhibition. These mechanisms can influence macrophage and T-cell polarization, cytokine programs, and endothelial oxidative stress responses. At the systemic level, SCFA-associated signaling has been linked to more favorable inflammatory profiles and endothelial vasodilatory function, consistent with a potential vasoprotective role in settings of low-grade inflammation. However, SCFA effects are context dependent: locally elevated concentrations within periodontal pockets may impair epithelial viability and exacerbate barrier disruption, thereby increasing exposure to inflammatory ligands at the lesion site (70). Thus, SCFAs can be viewed as a metabolite class with bidirectional effects, depending on compartment, concentration, and host inflammatory context (71, 72).

Trimethylamine-N-oxide (TMAO) has also been associated with vascular phenotypes relevant to atherothrombosis (73). Dietary precursors such as choline and carnitine can be converted by microbial pathways to trimethylamine (TMA), which is subsequently oxidized in the liver to TMAO. Experimental and clinical studies have linked higher TMAO exposure to endothelial activation programs, oxidative stress, foam cell biology, and platelet reactivity, and in some cohorts to increased cardiovascular risk (74). Although findings vary across populations and dietary patterns, these observations are directionally aligned with a role for TMAO as an amplifying factor within pro-inflammatory vascular pathways rather than a uniform, independent determinant of risk (75).

A further pathway connecting oral microecology to vascular function is the nitrate-nitrite-NO axis, which provides an alternative, non–eNOS-dependent source of NO and depends on oral nitrate-reducing bacteria (76). Oral reduction of dietary nitrate to nitrite enables downstream NO generation and may partially compensate for reduced eNOS-derived NO bioavailability under inflammatory conditions. Interventional and quasi-experimental observations indicate that suppression of oral nitrate-reducing communities (e.g., by antiseptic mouthwash use) reduces circulating nitrite and has been associated with higher blood pressure and impaired endothelium-dependent vasodilation (77, 78). These findings support a physiologic coupling between oral microbial function and systemic NO homeostasis (79, 80).

At the vascular interface, these metabolite-associated signals can converge on redox-sensitive endothelial programs that regulate eNOS coupling, NO bioavailability, and adhesion phenotypes, thereby modulating leukocyte recruitment propensity under inflammatory conditions. In humans, circulating metabolite measures (e.g., TMAO and SCFAs) have been associated with endothelial and vascular phenotypes in multiple settings, whereas longitudinal studies that integrate oral-resolved functional microecology with metabolite flux and within-person endothelial readouts remain relatively sparse (81). Collectively, these metabolite-linked pathways provide mechanistically plausible routes by which oral microecology may modulate systemic inflammatory thresholds and endothelial function, particularly via redox balance and NO signaling. A rigorous near-term strategy is to evaluate metabolite measures (e.g., SCFAs, TMAO, nitrite-related readouts) alongside endothelial and inflammatory phenotypes in longitudinal human cohorts and mechanistic intervention studies, with explicit attention to heterogeneity across oral microecology and immune phenotypes (71, 73, 79).

## 4 Controversies, challenges, and knowledge gaps

As outlined above, population-level signals and mechanistic observations are directionally consistent, supporting biological plausibility and cross-scale replicability of the oral–vascular axis. However, current evidence falls short of establishing a closed causal chain. Animal and cell studies mainly provide feasibility and path signals; RCTs on hard endpoints remain limited; Mendelian randomization (MR) findings are inconsistent or show small effects. From the standpoint of association rather than causality, we focus on four key questions and testable paths.

### 4.1 Causality remains unproven

Mechanistically, *P. gingivalis* and its OMVs can induce eNOS inhibition, increased endothelial permeability, and inflammatory amplification in atheroprone animals and endothelial models; blockade of TLR–NF-κB pathways partially attenuates these phenotypes, suggesting a feasible route by which *P. gingivalis* or its OMVs trigger endothelial dysfunction (66–68). Experimental periodontitis is also associated with trained immunity and hematopoietic bias at the level of hematopoietic stem and progenitor cells, and such central trained immunity has been shown to be transferrable in animals, providing indirect evidence for a cross-organ link between oral inflammation, bone marrow reprogramming, and systemic inflammatory set-points (56, 82, 83).

Clinically, existing randomized evidence has not established that periodontal therapy reduces hard endpoints such as MI, stroke, or cardiovascular death; positive signals remain largely confined to surrogate vascular markers (e.g., FMD, cIMT), and extrapolation to events should therefore be cautious (84). This boundary is consistent with systematic evidence syntheses concluding that, in people with established CVD, there is no reliable trial evidence to confirm benefit on cardiovascular outcomes (85). Even at the surrogate level, effect sizes appear modest and heterogeneous: intensive periodontal treatment can induce acute, short-term systemic inflammation with transient worsening of endothelial function, followed by improvement at later follow-up (86), indicating that timing, protocol intensity, and maintenance strategies can materially affect direction and magnitude. Such heterogeneity, together with limited sample sizes, follow-up duration, variable stratification and intervention intensity, divergent maintenance, and dilution from concurrent cardiovascular risk management, constrains causal interpretation and likely contributes to small pooled effects in some meta-analytic summaries (20, 35).

In parallel, MR studies have not produced a consistent positive causal signal for major ASCVD outcomes; large two-sample MR analyses report no robust evidence that genetic liability to periodontitis causally increases coronary artery disease or stroke risk (19), and recent syntheses similarly emphasize predominantly null genetic-causality signals (87). These null results may reflect true independence, but they are also compatible with phenotype heterogeneity (case definition/severity misclassification) and weak-instrument limitations that bias estimates toward the null, particularly if effects are subgroup-specific or mediated through intermediate inflammatory pathways (88).

Thus, current data support feasible mechanisms and robust associations rather than proven causality. Priority should be given to event-driven, adequately powered, multi-center RCTs with strong interventions and standardized maintenance, complemented by high-quality MR, mediation analysis, and causal directed acyclic graphs (DAGs), in order to test reversibility and specificity at the population level.

### 4.2 Identifying and handling confounding

Periodontitis and CVD share numerous common causes (smoking, glycemic dysregulation, obesity, socioeconomic status). Even with strict multivariable adjustment and propensity-score matching, unmeasured or mismeasured confounders—such as long-term oral hygiene behaviors, healthcare access, and dietary patterns—are difficult to fully eliminate, leaving the association vulnerable to confounding (19, 89).

A more robust strategy combines repeated exposure and outcome measurement in prospective cohorts, negative control outcomes or exposures, and complementary tools such as E-values, instrumental variable analysis, MR, sensitivity analyses, and multilevel modeling to assess robustness (90, 91). In interventional studies, smoking cessation, glycemic control, and periodontal maintenance frequency should be prespecified as co-interventions and covariates to reduce dilution. Together, these approaches may help delineate the independent contribution of the oral–vascular axis (9, 92).

### 4.3 Complexity of the microbiome

Growing evidence supports a polymicrobial synergy and dysbiosis (PSD) paradigm in periodontitis, in which pathogenicity reflects community-level function rather than single-species effects. In this framework, Porphyromonas gingivalis may act as a low-abundance “keystone” amplifier that reshapes interaction networks and host immune thresholds, thereby increasing the abundance and systemic availability of functional outputs—pro-inflammatory ligands, OMVs, and metabolites—more directly than it determines community composition per se (93–96).

Two functional examples illustrate how oral communities can tune host sensing beyond taxonomic profiles. First, *P. gingivalis* LPS exhibits lipid A structural heterogeneity; tetra- versus penta-acylated lipid A variants differ in TLR-dependent inflammatory signaling, providing a mechanism by which the same organism can modulate innate activation intensity across microenvironmental states (97). Second, *P. gingivalis* OMVs provide a selective export route for virulence determinants: proteomic studies show enrichment of multiple virulence-associated proteins, including gingipains, and OMVs can induce gingipain-dependent increases in vascular permeability in experimental systems, supporting mechanistic feasibility for vesicle-mediated delivery to endothelial interfaces even when sustained bacterial viability at target sites is uncertain (98, 99).

A repeatedly reported metabolite axis linked to vascular phenotypes is the microbe–host TMA/TMAO pathway. In clinical cohorts, higher circulating TMAO has been associated with cardiovascular risk, and experimental studies are directionally aligned with pro-atherothrombotic biology; periodontal studies further suggest elevated TMAO in advanced disease and correlations with endothelial dysfunction indices, while remaining vulnerable to confounding by diet and cardiometabolic comorbidity (100–102). Finally, although oral longitudinal multi-omics studies demonstrate coordinated shifts in plaque functional potential, metabolite profiles, and host inflammatory mediators across gingival states (13), integrated human designs that link oral-resolved functional microecology to metabolite flux and within-person endothelial phenotyping remain limited. These gaps motivate longitudinal and interventional studies that co-measure oral functional ecology (including ligand structure and OMV cargo), systemic metabolite axes, and harmonized vascular endpoints under explicit evidence-tier language.

### 4.4 Host genetic susceptibility

Periodontitis has a heritable component, but recent genome-wide association study (GWAS)/transcriptome-wide association study (TWAS) remain limited by sample size, phenotype harmonization, and multi-ancestry replication, resulting in relatively few robust loci and inconsistent or small shared genetic architecture with coronary disease (82, 83). A key contributor is phenotype heterogeneity: clinically adjudicated staging/extent is not uniformly available at scale, and commonly used proxy definitions can have low single-nucleotide polymorphism (SNP)-heritability, reducing power for cross-trait analyses even when large cohorts are available (103).

Shared genetic architecture with coronary artery disease (CAD) has nevertheless been formally interrogated using genome-wide methods. For example, cross-trait linkage disequilibrium (LD) score regression in a Mendelian-randomization framework reported no statistically significant genetic correlation between periodontitis and CAD (rg ≈ 0.10; p ≈ 0.43), consistent with at most modest polygenic overlap under current phenotype definitions and instrument strength (19). At the same time, locus-level overlap has been described, including a shared association signal at 9p21.3 (antisense noncoding RNA in the INK4 locus [ANRIL]/CDKN2A/CDKN2B regulatory region) for (CHD) and aggressive periodontitis, as well as a GWAS meta-analytic approach identifying CAD loci (e.g., vesicle-associated membrane protein 8 [VAMP8]; a disintegrin and metalloproteinase with thrombospondin motifs 7 [ADAMTS7]) that also associate with periodontitis with concordant direction and small effect sizes (104, 105). Cross-phenotype prediction has also been tested: in UK Biobank, a genome-wide periodontal disease polygenic risk score showed a modest association with CAD risk (per standard deviation [SD] increase OR ≈ 1.03, 95% CI ≈ 1.02–1.05), supporting partial sharing while underscoring limited standalone predictive utility (106).

Overall, the current evidence is most compatible with partial, phenotype-sensitive overlap rather than a strong shared genetic architecture, and apparent null results plausibly reflect a mixture of low power (weak instruments/low heritability in proxies), phenotype heterogeneity, and true independence for portions of the causal graph. Accordingly, a more realistic goal is to use genetic markers for risk stratification and mechanistic linkage—integrated with inflammatory phenotypes, endothelial function, and hematopoietic bias—to clarify which individuals are more susceptible to the oral–vascular axis and under what conditions (103, 107).

## 5 Translational medicine and future perspectives

Evidence around the oral–vascular axis is evolving from purely associative and mechanistic signals toward measurable, stratifiable, and actionable clinical pathways. This section focuses on biomarker development, precision stratification, novel interventions, and interdisciplinary collaboration.

### 5.1 Biomarkers

Serum antibodies against *P. gingivalis*—including immunoglobulin G (IgG)/immunoglobulin A (IgA) directed at major virulence factors—have repeatedly been associated with atherosclerotic phenotypes and CVD status in observational settings, supporting their use as candidate indicators of host–microbe immune exposure within the oral–vascular axis (2, 108, 109). However, association does not establish clinical utility. Evidence remains limited regarding whether periodontal serology improves prediction beyond established ASCVD risk models (e.g., discrimination, calibration, and clinically meaningful reclassification), and large prospective, multi-center external validations with prespecified endpoints are lacking. Accordingly, no antibody-based biomarker currently meets criteria for routine clinical integration, and these measures should be framed primarily as research tools for mechanistic phenotyping and cohort enrichment rather than as actionable clinical tests (110, 111).

ACPAs are frequently elevated at the intersection of periodontitis and vascular inflammation and may reflect subgroup-relevant immune activation, particularly in rheumatoid arthritis contexts (88). In non-RA populations, however, assay standardization, thresholds, intra-individual variability, and—critically—incremental predictive value beyond standard risk factors remain insufficiently defined for clinical use (112).

More broadly, functional oral microecological indicators (e.g., OMV burden, LPS structural variants, diffusible metabolite profiles) are conceptually attractive because they may more directly represent biologically active exposures than species-based metrics (94, 113, 114). Nonetheless, translation requires a staged evidentiary pipeline separating analytical validity, clinical validity, and clinical utility (context of use), consistent with the United States Food and Drug Administration (FDA)/National Institutes of Health (NIH) Biomarkers, EndpointS, and other Tools (BEST) framework. For risk prediction, improvements in net reclassification improvement (NRI)/IDI should be treated cautiously: these metrics can be unstable or misleading without prespecified risk categories, rigorous external validation, and demonstrated decision impact (115). Current American College of Cardiology/American Heart Association prevention guidance also underscores that only well-validated “risk enhancers” should inform clinical risk assessment, reinforcing that periodontal antibody or oral-function biomarkers should not be positioned for implementation until late-phase validation demonstrates reproducible incremental value and net clinical benefit (116).

### 5.2 Precision stratification

One testable strategy is two-dimensional stratification based on oral microecological functional profiles and systemic immuno-inflammatory phenotypes to identify populations more sensitive to the oral–vascular axis. For example, individuals with higher baseline *P. gingivalis*–related antibody titers or elevated oral and plasma OMV indices, combined with elevated systemic inflammatory markers, may be more prone to endothelial dysfunction or accelerated cIMT progression. This strategy requires prospective cohorts with longitudinal multi-omics and vascular phenotyping, with explicit consideration of shared risk factors such as smoking and metabolic abnormalities to reduce confounding (114).

A hypothetical trial design might enroll patients with moderate-to-severe periodontitis, hs-CRP ≥2 mg/L or neutrophilia, and *P. gingivalis* antibody or OMV indices in the upper tertile. Primary endpoints could include changes in FMD and annual cIMT progression over 12–24 months; secondary endpoints could be composite cardiovascular events (event-driven). This is proposed as a template for research design rather than clinical guidance.

### 5.3 Interventional strategies

Translational interventions are moving from broad-spectrum antimicrobial approaches to function-centered strategies that suppress key virulence axes, reshape microecological networks, and modestly modulate shared inflammatory pathways while preserving commensal stability.

As an example of virulence inhibition, *P. gingivalis* cysteine proteases (gingipains) sit upstream of tissue destruction and immune modulation. Preclinical and pharmacological studies suggest that small-molecule and peptide gingipain inhibitors are biologically feasible targets, but cardiovascular outcome data are lacking, and such agents are currently more appropriate as developmental candidates than routine clinical tools (117, 118).

In parallel, microecological interventions emphasize minimal disturbance, precise targeting, and optimized local delivery to reduce impact on commensals and resistance pressure. As adjuncts to NSPT, probiotics and synbiotics have been associated with consistent improvements in clinical parameters and inflammatory markers in small-to-medium RCTs and recent systematic and network meta-analyses, but effect sizes are influenced by population heterogeneity and maintenance frequency. Larger, standardized, longer-term trials are needed to evaluate durability and generalizability (119–121).

On the immune pathway side, IL-1β inhibition in secondary cardiovascular prevention (Canakinumab Anti-inflammatory Thrombosis Outcomes Study (CANTOS) trial) reduced MACE in patients with prior MI and elevated inflammation, demonstrating that inflammatory pathways are targetable (122). However, these data were not obtained in periodontitis cohorts, and extrapolation to patients with high periodontal burden and systemic inflammation requires event-driven randomized trials to assess safety and benefit–risk, ideally alongside standardized periodontal care.

### 5.4 Interdisciplinary collaboration

Integrating dental expertise into multidisciplinary cardiovascular care teams may improve coordination of care (particularly through bidirectional referral pathways, shared decision-making, and harmonized management of common risk factors) without implying that periodontal screening should be adopted as a validated component of cardiovascular risk assessment. Scoping reviews and health-services research suggest that collaborative models can improve process measures (e.g., access, continuity, adherence, and preventive-care delivery), but evidence for reductions in MI, stroke, or cardiovascular mortality, as well as robust cost-effectiveness evaluations, remains limited and has not yet been demonstrated in event-driven trials. Accordingly, current evidence supports periodontal treatment on established oral-health indications, whereas any cardiovascular benefit should be regarded as investigational pending adequately powered RCTs and prospective implementation studies with standardized endpoints, pre-specified care pathways, and interoperable information systems to enable rigorous evaluation (123).

## 6 Conclusions and outlook

The oral cavity is not an isolated organ but an important mucosal ecological niche that contributes to systemic homeostasis through continuous host–microbe immune crosstalk. Framed around the oral–vascular axis, this review synthesizes epidemiologic associations, key immunopathological mechanisms, and translational perspectives linking oral diseases, particularly periodontitis, to distant vascular diseases.

Large scale cohorts and meta analyses show a stable, reproducible association between periodontitis and atherosclerotic cardiovascular events, with evidence of dose response and residual risk after adjustment for traditional factors. However, the strength, directionality, and reversibility of this association at the causal level remain to be established by event driven randomized trials and rigorous causal inference.

Mechanistically, the oral–vascular axis can be framed as a spatiotemporal, dual-regulatory network in which immune activation and immunometabolic remodeling may jointly shape vascular susceptibility. Local periodontal inflammation can increase systemic exposure to microbial ligands and vesicular cargo, providing recurrent pattern-recognition and antigenic stimulation, while inflammatory spillover and acute-phase responses may interact with myeloid priming consistent with trained immunity to elevate systemic inflammatory set-points. These processes are plausibly linked to endothelial activation and reduced nitric oxide bioavailability, which may lower the threshold for leukocyte recruitment, arterial remodeling, and plaque progression. In this framework, endothelial dysfunction and sustained innate/adaptive immune activation can be viewed as part of a continuum from potentially reversible functional perturbation to structural lesion development and, in susceptible contexts, thromboinflammatory complications. Functional variation in oral microecology and host susceptibility factors—including genetic background and immune phenotype—may contribute to inter-individual differences in immune tone, vascular phenotypes, and therapeutic responsiveness.

Despite rapidly accumulating mechanistic evidence, the most cautious current interpretation is that the relationship between periodontitis and CVD is biologically plausible and cross scale consistent but not yet proven causal. Key challenges are to quantify the independent contribution of oral driven immune activation amid complex confounding, to define which immune circuits are necessary and sufficient for vascular effects, and to pursue clinical translation without overstating the evidence.

Future work should advance along complementary lines that preserve evidentiary rigor and avoid premature clinical extrapolation: well-designed prospective cohorts and event-driven trials, ideally with repeated measures of periodontal exposure, vascular outcomes, and immune/endothelial phenotypes, are needed to test temporal ordering, reversibility, specificity, and potential immunologic mediation; in parallel, integrated functional omics spanning microbial activity and host immunity can help identify reproducible and quantifiable biomarkers and mechanistically grounded targets for stratification and monitoring, including signatures consistent with trained immunity and immunometabolic remodeling; finally, carefully evaluated models of interdisciplinary exchange between dental and cardiovascular services may improve coordination and hypothesis-driven phenotyping, but any extension toward co-management or immunology-informed cardiovascular risk assessment should remain explicitly investigational until validated by adequately powered trials and implementation studies with prespecified endpoints and cost-effectiveness analyses.

The oral–vascular axis offers an integrative immunological perspective for understanding how chronic mucosal inflammation may shape vascular immunopathology and opens new interfaces among cardiovascular prevention, oral health, and immunology. By acknowledging current limitations while closing the loop between mechanisms, population data, and clinical trials, future studies can test whether targeting oral driven immune dysregulation yields actionable clinical strategies and measurable public health gains.

## Glossary

AAP: American Academy of Periodontology
ACPA: anti-citrullinated protein antibody
ADAMTS7: a disintegrin and metalloproteinase with thrombospondin motifs 7
ANRIL: antisense noncoding RNA in the INK4 locus
ARIC: Atherosclerosis Risk in Communities Study
ASCVD: atherosclerotic cardiovascular disease
ApoA-I: apolipoprotein A-I
BEST: Biomarkers, EndpointS, and other Tools
C5a: complement component 5a
C5aR: complement component 5a receptor
CAD: coronary artery disease
CAL: clinical attachment loss
CANTOS: Canakinumab Anti-inflammatory Thrombosis Outcomes Study
CDC: Centers for Disease Control and Prevention
CHD: coronary heart disease
CI: confidence interval
CRP: C-reactive protein
CTLA-4: cytotoxic T-lymphocyte–associated protein 4
CVD: cardiovascular disease
cIMT: carotid intima–media thickness
DAG: directed acyclic graph
DAMP: damage-associated molecular pattern
DNA: deoxyribonucleic acid
eNOS: endothelial nitric oxide synthase
FDA: United States Food and Drug Administration
FcγR: Fc gamma receptor
FMD: flow-mediated dilation
GBD: Global Burden of Disease
GPR41: G protein–coupled receptor 41
GPR43: G protein–coupled receptor 43
GWAS: genome-wide association study
H3K27ac: histone H3 lysine 27 acetylation
H3K4me3: histone H3 lysine 4 trimethylation
HDL: high-density lipoprotein
HR: hazard ratio
hs-CRP: high-sensitivity C-reactive protein
ICAM-1: intercellular adhesion molecule 1
IFN-γ: interferon-γ
IgA: immunoglobulin A
IgG: immunoglobulin G
IL-1β: interleukin-1β
IL-6: interleukin-6
IL-18: interleukin-18
IPT: intensive periodontal therapy
IRR: incidence rate ratio
LCAT: lecithin–cholesterol acyltransferase
LD: linkage disequilibrium
LPS: lipopolysaccharide
LtxA: leukotoxin A (Aggregatibacter actinomycetemcomitans leukotoxin)
MAPK: mitogen-activated protein kinase
MCP-1: monocyte chemoattractant protein 1
MI: myocardial infarction
MR: Mendelian randomization
MyD88: myeloid differentiation primary response 88
NADPH: nicotinamide adenine dinucleotide phosphate
NET: neutrophil extracellular trap
NF-κB: nuclear factor κB
NHANES: National Health and Nutrition Examination Survey
NHLBI: National Heart, Lung, and Blood Institute
NIH: National Institutes of Health
NLRP3: NLR family pyrin domain containing 3
NOD: nucleotide-binding oligomerization domain
NO: nitric oxide
NRI: net reclassification improvement
NT-proBNP: N-terminal pro–B-type natriuretic peptide
OMVs: outer membrane vesicles
OR: odds ratio
PAF: population attributable fraction
PAMP: pathogen-associated molecular pattern
PD-1: programmed cell death protein 1
PD-L1: programmed death-ligand 1
pH: potential of hydrogen
PPAD: Porphyromonas gingivalis peptidylarginine deiminase
PPC: periodontal profile class
PRR: pattern-recognition receptor
RA: rheumatoid arthritis
RCT: randomized controlled trial
RNA: ribonucleic acid
RR: risk ratio (relative risk)
ROS: reactive oxygen species
SAA: serum amyloid A
SCFA: short-chain fatty acid
SD: standard deviation
SEV: standard error of the variance
SHIP-START: Study of Health in Pomerania (SHIP), START cohort (SHIP-START)
SNP: single-nucleotide polymorphism
TLR: Toll-like receptor
TLR2/4: Toll-like receptors 2/4
TMA: trimethylamine
TMAO: trimethylamine-N-oxide
TNF-α: tumor necrosis factor-α
TWAS: transcriptome-wide association study
VCAM-1: vascular cell adhesion molecule 1
VAMP8: vesicle-associated membrane protein 8
ZO-1: zonula occludens 1
